# Cytosolic re-localization and optimization of valine synthesis and catabolism enables inseased isobutanol production with the yeast *Saccharomyces cerevisiae*

**DOI:** 10.1186/1754-6834-5-65

**Published:** 2012-09-06

**Authors:** Dawid Brat, Christian Weber, Wolfram Lorenzen, Helge B Bode, Eckhard Boles

**Affiliations:** 1 Institute of Molecular Biosciences, Goethe-University Frankfurt, Max-von-Laue-Str. 9, 60438, Frankfurt am Main, Germany

**Keywords:** Isobutanol, *Saccharomyces*, Fermentation, Valine biosynthesis, Ehrlich pathway, Yeast, Genetic engineering, Biofuel, Butanol

## Abstract

**Background:**

The branched chain alcohol isobutanol exhibits superior physicochemical properties as an alternative biofuel. The yeast *Saccharomyces cerevisiae* naturally produces low amounts of isobutanol as a by-product during fermentations, resulting from the catabolism of valine. As *S. cerevisiae* is widely used in industrial applications and can easily be modified by genetic engineering, this microorganism is a promising host for the fermentative production of higher amounts of isobutanol.

**Results:**

Isobutanol production could be improved by re-locating the valine biosynthesis enzymes Ilv2, Ilv5 and Ilv3 from the mitochondrial matrix into the cytosol. To prevent the import of the three enzymes into yeast mitochondria, N-terminally shortened Ilv2, Ilv5 and Ilv3 versions were constructed lacking their mitochondrial targeting sequences. SDS-PAGE and immunofluorescence analyses confirmed expression and re-localization of the truncated enzymes. Growth tests or enzyme assays confirmed enzymatic activities. Isobutanol production was only increased in the absence of valine and the simultaneous blockage of the mitochondrial valine synthesis pathway. Isobutanol production could be even more enhanced after adapting the codon usage of the truncated valine biosynthesis genes to the codon usage of highly expressed glycolytic genes. Finally, a suitable ketoisovalerate decarboxylase, Aro10, and alcohol dehydrogenase, Adh2, were selected and overexpressed. The highest isobutanol titer was 0.63 g/L at a yield of nearly 15 mg per g glucose.

**Conclusion:**

A cytosolic isobutanol production pathway was successfully established in yeast by re-localization and optimization of mitochondrial valine synthesis enzymes together with overexpression of Aro10 decarboxylase and Adh2 alcohol dehydrogenase. Driving forces were generated by blocking competition with the mitochondrial valine pathway and by omitting valine from the fermentation medium. Additional deletion of pyruvate decarboxylase genes and engineering of co-factor imbalances should lead to even higher isobutanol production.

## Background

Biofuels produced from renewable resources are an attractive alternative to supplement or replace fossil fuels. Currently, bioethanol represents the most prominent biofuel obtained by microbial fermentation. However, compared to ethanol 'higher' alcohols have several advantages as alternative biofuels [1].

**Figure 1:**
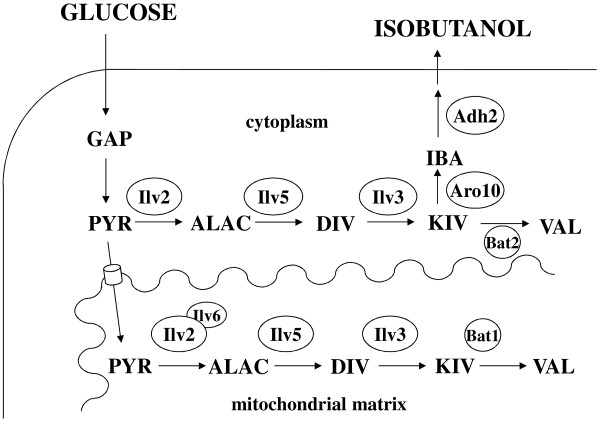
**Schematic illustration of the synthetic isobutanol biosynthesis pathway.** Glucose is converted to pyruvate via glycolysis. Pyruvate can be further converted to 2-ketoisovalerate (KIV) in the cytosol by the re-localized Ilv2, Ilv5 and Ilv3 enzymes. KIV is metabolized into isobutanol via the Ehrlich pathway reactions catalyzed by Aro10 and Adh2. GAP = glyceraldehyde-3-phosphate; PYR = pyruvate; ALAC = 2-acetolactate; DIV = 2,3-dihydroxyisovalerate; IBA = isobutyraldehyde.

Isobutanol is a normal by-product of yeast fermentations, but only in very small amounts [2,3]. It can be synthesized via a three-step catalytic breakdown of valine, the so-called Ehrlich pathway [3,4]. Thereby, valine undergoes transamination to 2-ketoisovalerate (KIV) catalyzed by branched-chain amino acid aminotransferase (Bat2). The subsequent decarboxylation and reduction of KIV to isobutanol is catalyzed by ketoacid decarboxylase (KDC) and alcohol dehydrogenase (ADH) with isobutyraldehyde as an intermediate. KIV is also an intermediate of the *de novo* synthesis of valine and is thus a common intermediate of both, valine synthesis and degradation (Figure 1) [5]. The enzymes which provide KIV by *de novo* synthesis are acetolactate synthase (Ilv2), acetohydroxyacid reductoisomerase (Ilv5) and dihydroxyacid dehydrates (Ilv3) [5]. These enzymes convert pyruvate to KIV by condensation of two molecules of pyruvate to 2-acetolactate (ALAC) and CO_2_, reduction of ALAC to 2,3-dihydroxyisovalerate (DIV) and dehydratation to KIV. The conversion of KIV to valine is finally catalyzed by branched-chain amino acid aminotransferase (Bat1) [6].

The coupling of valine biosynthetic enzymes with valine degrading enzymes via the common intermediate KIV would result in a direct isobutanol synthesis pathway. Such a strategy could be successfully transferred into different bacterial microorganisms. In various recent publications, the metabolic flux towards isobutanol production was improved by overexpressing endogenous or heterologous genes of valine synthesis and degradation. E.g., engineered recombinant *E. coli* strains were able to produce more than 20 g/L isobutanol, whereby isobutanol amounts could be further enhanced up to 50 g/L by using a 1 L bioreactor connected to a gas-stripping system [7,8]. Production of isobutanol with *Bacillus subtilis* and *Corynebacterium glutamicum* could be achieved up to 2.62 g/L and 4.9 g/L, respectively [9,10].

One of the major problems of most bacterial host organisms in large production processes is their low tolerance towards fermentation inhibitors and to isobutanol [1]. The yeast *S. cerevisiae* seems to be more promising as a host for isobutanol production [1]. Previous work has demonstrated that *S. cerevisiae* possesses beneficial properties such as higher tolerance towards butanol and a high robustness against toxic inhibitors and fermentation products. Additionally, fermentations are performed at low pH values, whereby the risk of contaminations is minimized [1]. Traditionally, *S. cerevisiae* is used already since centuries in applications like beer brewing or industrial ethanol production.

Recently, enhanced isobutanol production by *S. cerevisiae* has first been demonstrated by overexpression of the endogenous genes involved in valine metabolism. The recombinant strain produced isobutanol with a maximum yield of 4.12 mg isobutanol/g glucose [11]. In another work the final titer was increased up to 143 mg/L at a yield of 6.6 mg/g glucose by overexpressing in a Δ*pdc1* deletion strain the first gene of valine biosynthesis (*ILV2,* encoding acetolactate synthase) and genes encoding enzymes catalyzing the degradation of KIV (*kivD* of *Lactococcus lactis* and *ADH6* of *S. cerevisiae*) [12].

In contrast to bacteria, in the yeast *S. cerevisiae* anabolic reactions providing KIV are separated from catabolic reactions producing isobutanol. The anabolic reactions are part of valine biosynthesis and are located in the mitochondrial matrix, whereas the Ehrlich pathway reactions take place in the cytosol [13,14]. We hypothesized that the presence of all the enzymes within the same compartment would presumably increase the production of isobutanol. Due to the loss of mitochondrial function and inaccessibility of mitochondrially located enzymes at high glucose concentrations or during anaerobic conditions, a cytosolic localization of the new isobutanol synthesis pathway seemed to be very promising. Moreover, this would also avoid any transport of intermediates across intracellular membranes. Therefore, we aimed to re-localize the enzymes of valine biosynthesis from the mitochondrial matrix into the cytosol (Figure 1). These enzymes are synthesized as precursor proteins containing an N-terminal mitochondrial targeting sequence (MTS) [5,15,16]. During translocation into the mitochondrial matrix, the N-terminal presequence is cleaved off by a mitochondrial specific processing peptidase [17]. Therefore, expression of N-terminally truncated enzyme versions lacking the MTS should lead to a cytosolic location. Indeed, in a recent work, overexpression of N-terminally truncated *ILV2ILV5* and *ILV3* together with overexpression of *Lactococcus lactis* KDC gene *kivD* resulted in a production of up to 151 mg/L isobutanol [18].

In our work we found that overexpression of cytosolically located Ilv2, Ilv5 and Ilv3 enzymes did not significantly increase isobutanol production. However, elimination of the competing mitochondrial valine pathway together with the omission of valine from the fermentation medium resulted in strongly increased isobutanol production. Finally, the highest titers were obtained after adaptation of the codon usage of valine biosynthetic genes to the glycolytic codon usage and additional overexpression of a suitable yeast KIV decarboxylase and a yeast isobutanol dehydrogenase.

## Results

### Disruption of the mitochondrial targeting sequences of the valine biosynthesis enzymes

**Figure 2:**
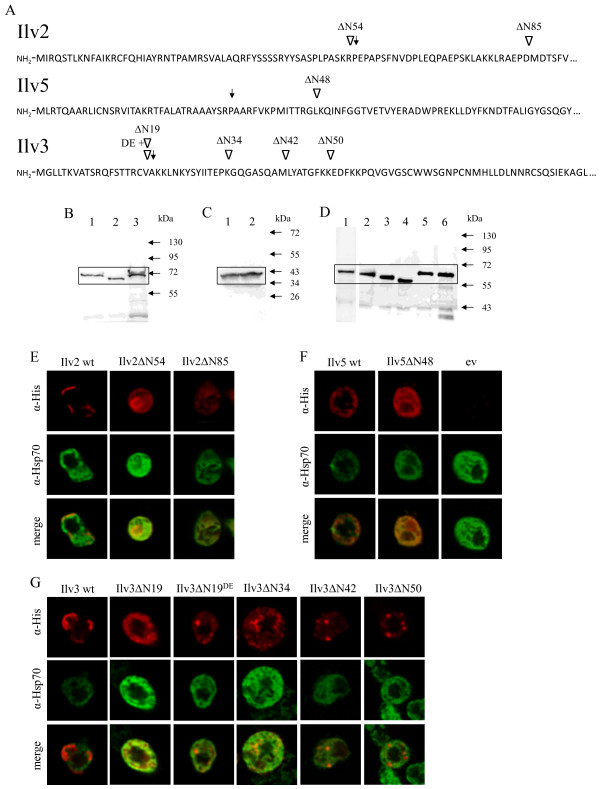
**Cytosolic re-localization of the isobutanol synthesis enzyms Ilv2, Ilv5 and Ilv3.** (**A**) N-terminal amino acid sequences of the precursor proteins Ilv2, Ilv5 and Ilv3. The N-termini of enzymes Ilv2, Ilv5 and Ilv3 were shortened to eliminate the N-terminal import signal sequences. Different truncations were constructed which were based on alignments with bacterial homologues and are indicated by inverted triangles. Truncations predicted by Mitoprot analysis are indicated by arrows. (**B**-**D**) Western blot analyses of wild-type and N-terminally truncated Ilv2, Ilv5 and Ilv3 proteins carrying a C-terminal 6His-tag. CEN.PK2-1C cells containing overexpression plasmids for the different proteins were grown on selective SCD media into the exponential growth phase, crude extracts were prepared and subjected to Western blot analyses. Bands of interest are framed. Panel B: Lane1: Ilv2ΔN54; Lane2: Ilv2ΔN85; Lane3: wild-type Ilv2. Panel C: Lane1: Ilv5ΔN48; Lane2: wild-type Ilv5. Panel D: Lane1: Ilv3ΔN19; Lane2: Ilv3ΔN34; Lane3: Ilv3ΔN42; Lane4: Ilv3ΔN50; Lane5: Ilv3ΔN19^DE^; Lane6: wild-type Ilv3. (**E**-**G**) Indirect immunofluorescence microscopy of wild-type and N-terminally truncated proteins carrying a C-terminal 6His-tag. Yeast cells as under B-D were grown on selective SCD media into the exponential growth phase, harvested and prepared as described in Material and Methods. α-His antibodies were applied for the visualisation of Ilv enzymes, α-Hsp70 antibodies for cytosolic staining. Panel E: localization of Ilv2 variants. Panel F: localization of Ilv5 variants and empty vector (ev). Panel G: localization of Ilv3 variants.

Isobutanol is a common by-product of yeast fermentations. However, isobutanol levels are very low and are dependent on the fermentation conditions [2,11]. Isobutanol derives from the degradation of valine via the Ehrlich pathway which takes place in the cytosol [2,19]. On the other hand, the biosynthesis of valine from pyruvate occurs in the mitochondria [13]. In order to re-locate Ilv2, Ilv5 and Ilv3 into the cytosol we wanted to overexpress these enzymes without their N-terminal mitochondrial targeting sequences. Mitochondrial targeting sequences are not clearly defined but have a length of typically 15-50 amino acids, forming positively charged amphipathic alpha helices [20]. Therefore, we tested different truncations for the individual enzymes. The choice for the truncated version of Ilv5 was based on previously published results [15] whereas the truncated versions of Ilv2 and Ilv3 were derived from alignments with bacterial homologues which do not possess mitochondrial import sequences (Figure 2A). Furthermore, the Mitoprot program was used for validation [21]. In the case of Ilv3ΔN19, additionally the version Ilv3ΔN19^DE^ was included adding the negatively charged amino acids D and E after the initial methionine to disturb any random positive charges.

The wild-type as well as various truncated ORF versions of *ILV2*, *ILV3* and *ILV5* were cloned via homologous recombination into multicopy overexpression plasmids with and without a six-histidin (6His)-tag at their C-termini. After transformation into CEN.PK2-1C the (6His)-tagged versions were used to verify in Western blot analyses that all truncated enzymes were indeed expressed with their expected sizes (Figure 2B-D).

### Localization of truncated Ilv enzymes by indirect immunofluorescence microscopy

To test the localization of the truncated Ilv enzymes, indirect immunofluorescence microscopy analyses were performed. Localization of the truncated enzymes was compared with their mitochondrial wild-type counterparts and the cytosolic marker protein Hsp70 (Figure 2E-G).

The Ilv2ΔN54 variant was clearly re-localized out of the mitochondria, most probably into the cytosol, although it seemed to cluster in a specific, undefined region of the cells (Figure 2E). The localization of Ilv2ΔN85 was difficult to determine but it was also not homogeneously distributed within the cells. Ilv5ΔN48 clearly co-localized with the cytosolic marker protein as already shown before (Figure 2F) [15]. In the case of Ilv3, the Ilv3ΔN19 variant was found to be in the cytosol although some prominent punctuated patterns could be observed which might indicate aggregation of the protein (Figure 2G). All the other truncated Ilv3 variants including Ilv3ΔN19^DE^ showed an increasing tendency to accumulate in these punctuated patterns.

### Complementation tests with truncated Ilv enzyme versions in their respective single deletion mutants

To analyze the properties of the truncated Ilv enzymes, complementation tests were performed in single *ilv* deletion strains by growth tests on media lacking valine or isoleucine. Deletion mutants were constructed for *ilv2*, *ilv5* and *ilv3* in strain CEN.PK2-1C, resulting in strains Isoy8, Isoy12 and Isoy10, respectively. The multicopy plasmids expressing the various truncated Ilv versions or the corresponding wild-type enzymes, with (not shown) or without C-terminal 6His-tags, as well as empty vector controls were transformed into the respective deletion mutants. If the individual truncated enzymes were re-localized out of the mitochondrial matrix into the cytosol but still were functional we expected to see at least a partial complementation of the growth defect depending on whether the metabolic intermediates are able to cross the mitochondrial membranes or not.

**Figure 3:**
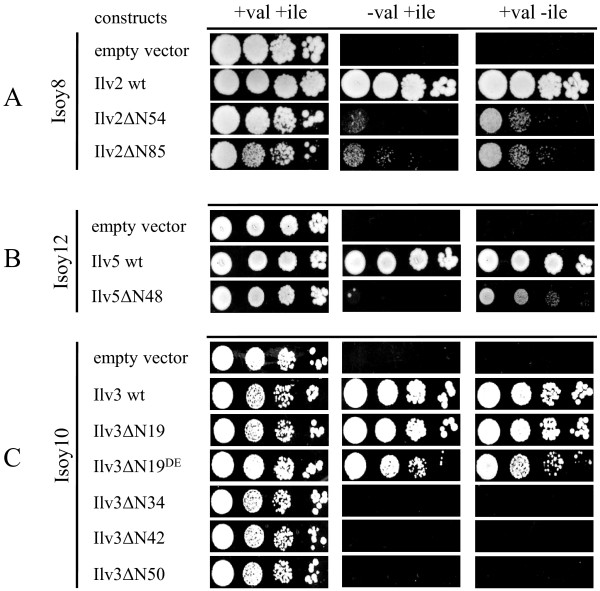
**Growth complementation analyses of single*ilv*deletion mutants.** Isoy8, Isoy10 and Isoy12 were transformed with plasmids overexpressing genes for wild-type or truncated Ilv2, Ilv3 and Ilv5 proteins. As a negative control strains were transformed with empty vectors. Transformants were grown in selective SCD medium, washed with sterile water and spotted in serial dilutions on selective SMD medium agar plates containing or lacking valine or isoleucine, respectively, and incubated for up to 7 days at 30°C. Panel **A**: Isoy8 containing empty vector or plasmids encoding wild-type Ilv2, Ilv5ΔN54 and Ilv2ΔN85, respectively. Panel **B**: Isoy12 containing empty vector or plasmids encoding wild-type Ilv5 and Ilv5ΔN48, respectively. Panel **C**: Isoy10 containing empty vector or plasmids encoding wild-type Ilv3, Ilv3ΔN19, Ilv3ΔN19^DE^, Ilv3ΔN34, Ilv3ΔN42 and Ilv3ΔN50, respectively.

For Ilv2 both truncated versions mediated very slow growth within seven days of incubation (Figure 3A). In the case of Ilv5ΔN48 only a few single colonies growing in the absence of valine could be observed (Figure 3B). However, when we tested complementation of the isoleucine auxotrophy of the *ilv5* strain by Ilv5ΔN48 faint growth of all the cells could be observed. For Ilv3 the truncated version Ilv3ΔN19 fully complemented the growth defect comparable to the wild-type enzyme and even the Ilv3ΔN19^DE^ version mediated fast growth in the absence of valine (Figure 3C). However, the other shortened Ilv3 versions did not complement the growth defect at all. Taken together with the localization analyses these results suggest that the truncated Ilv2 versions as well as the Ilv5ΔN48 and the Ilv3ΔN19 variants are active enzymes even outside the mitochondrial matrix.

To verify the enzyme activity of the Ilv5ΔN48 variant with 2-acetolactate, keto acid isomeroreductase enzymes assays were performed with crude extracts of the *ilv5* deletion strain Isoy12 transformed with plasmids overexpressing wild-type *ILV5*, *ILV5ΔN48* or empty vector. Extracts from cells overexpressing the wild-type *ILV5* gene catalyzed conversion of 2-acetolactate to 2,3-dihydoxyisovalerate at a maximal rate of 23.11 ± 2.04 mU mg protein^-1^ whereas the reaction in extracts derived from cells expressing *ILV5ΔN48* proceeded at a rate of 15.93 ± 1.77 mU mg protein^-1^. These results show that the truncated Ilv5 enzyme is nearly as active as the wild-type enzyme and suggest that its inability to complement growth of *ilv5* mutants in the absence of valine, but not of isoleucine, is only due to the failure of either its substrate 2-acetolactate (produced by mitochondrially localized Ilv2) to leave mitochondria or its product 2,3-dihydroxyisovalerate (needed by mitochondrially localized Ilv3) to enter mitochondria (or both) or to its low activity on 2-acetolactate.

### Re-localization of the whole valine biosynthesis pathway into the cytosol

**Figure 4:**
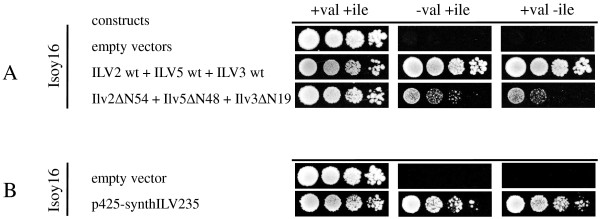
**Complementation experiments of triple*ilv*deletion mutants.** Isoy16 was transformed with plasmids expressing genes for wild-type and truncated Ilv2ΔN54, Ilv5Δ48 and Ilv3Δ19 enzymes, respective combinations, and codon-optimized genes for truncated Ilv2ΔN54, Ilv5Δ48 and Ilv3Δ19 proteins. Transformants were grown on selective SMD + leu-val + ile medium, washed with sterile water and spotted in serial dilutions on selective SMD medium agar plates containing or lacking valine or isoleucine, respectively, and incubated for up to 7 days at 30°C (detailed description in Methods). Cells containing empty vectors were pregrown on selective SMD + leu + val + ile medium. Panel **A**: Isoy16 containing empty vectors or plasmids encoding for wild-type or truncated Ilv2, Ilv5 and Ilv3, respective combinations. Panel **B**: Isoy16 expressing plasmid p425-synthILV235. Isoy16 transformed with empty vector were spotted as a negative control.

To test whether the combination of all three truncated re-localized Ilv enzymes could replace the mitochondrial valine biosynthesis pathway, a triple *ilv* deletion mutant (Δ*ilv2* Δ*ilv5* Δ*ilv3*; Isoy16) was constructed in CEN.PK2-1C. Plasmids expressing *ILV2ΔN54*, *ILV5ΔN48* and *ILV3ΔN19* were transformed together into this strain. As a negative control empty vectors and as a positive control plasmids overexpressing wild-type *ILV2*, *ILV5* and *ILV3* were also transformed, respectively. Cells expressing the re-localized Ilv enzymes could grow in the absence of valine or isoleucine although slightly slower than those with the wild-type enzymes (Figure 4A). These results suggest that the new cytosolic valine biosynthetic pathway can replace the native mitochondrial valine pathway of *S. cerevisiae*.

### Expression of codon-optimized truncated *ILV* genes

To increase Ilv protein expression for improved cytosolic KIV production, the codon usage of *ILV2ΔN54ILV5ΔN48* and *ILV3ΔN19* genes was adapted to that of the highly expressed glycolytic genes of *S. cerevisiae* but without changing the amino acid sequence. This approach has previously been shown to overcome bottlenecks in engineering of heterologous pathways and to improve sugar utilization in *S. cerevisiae* engineered for pentose fermentation [22,23]. In *ILV2ΔN54* 285 from 635 codons were changed, in *ILV5ΔN48* 67 from 349 and in *ILV3ΔN19* 223 from 568. All three truncated codon optimized *ILV*-ORFs were cloned on the same 2 μ multicopy plasmid and were placed between strong and constitutive glycolytic gene promoters and terminators, resulting in plasmid p425-synthILV235.

Strain Isoy16 was transformed with plasmid p425-synthILV235 and as negative control with an empty vector, respectively, and tested for valine and isoleucine prototroph. Cells expressing the codon-optimized version of the valine pathway grew even faster than those with the non-optimized truncated *ILV* genes (Figure 4B). The results suggest that codon-optimization clearly improved the flux through the new cytosolic valine pathway.

### Enhancement of Ehrlich pathway reactions

Basically, the complete isobutanol pathway should consist of three parts of different pathways: glycolysis to provide pyruvate, valine biosynthesis to metabolize pyruvate to KIV and the Ehrlich pathway which is required for degradation of KIV to isobutanol. Metabolization of KIV to isobutanol can be catalyzed by KDC-like and ADH enzymes [2,19] (Figure 1). Therefore, in order to complete the isobutanol pathway we investigated candidate enzymes useful for decarboxylation of KIV to isobutyraldehyde and reduction of isobutyraldehyde to isobutanol.

### KDC activity of Aro10

As KIV-decarboxylase (KDC) activity links valine metabolism and Ehrlich pathway, a high activity of this enzyme reaction is essential for high levels of isobutanol production. In *S. cerevisiae* five endogenous enzymes encoded by *PDC1PDC5PDC6ARO10* and *THI3* have been postulated to be involved in the decarboxylation of KIV to isobutyraldehyde [2,19]. The three Pdc enzymes are also involved in pyruvate decarboxylation in the ethanol fermentation pathway of yeast. As in a final industrial isobutanol producing yeast strain, the three *PDC* genes are important targets for blocking ethanol fermentation and as Thi3 activity contributes rather to leucine and isoleucine catabolism [19,24-27], we investigated the effects of overexpression of *ARO10*[28,29] and of the bacterial *kivD* of *Lactococcus lactis*[30].

**Figure 5:**
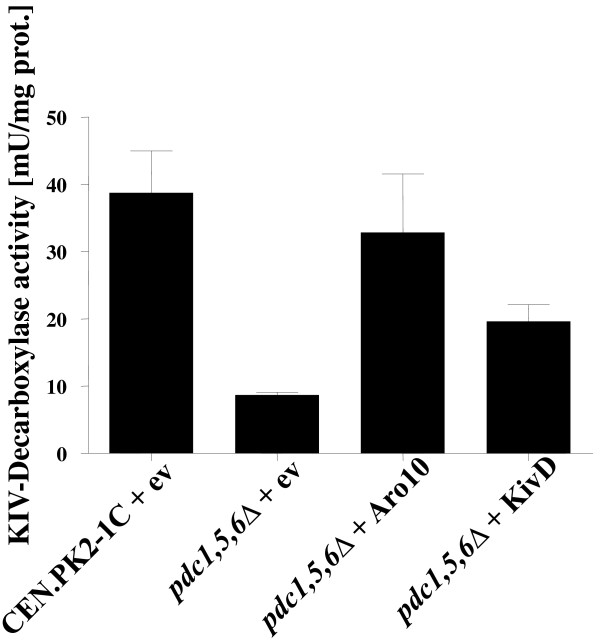
**KDC activities.** Strain Isoy21 was transformed with plasmids overexpressing *ARO10* of *S. cerevisiae*, *kivD* of *Lactococcus lactis* or the empty vector. The wild-type strain CEN.PK2-1C was transformed with the empty vector. Transformants were grown in selective SCD medium and KDC activity determined in crude extracts and normalized to the amount of protein.

To determine KDC activity of suitable enzymes, the strain Isoy21 (a CEN.PK2-1C Δ*pdc*^*-*^ suppressor strain) was transformed with overexpression plasmids encoding Aro10, KivD or the empty vector, respectively. Additionally the wild-type strain CEN.PK2-1C was transformed with an empty vector. Overexpression of *ARO10* in strain Isoy21 could nearly fully substitute the KDC activity of Pdc enzymes (32.83 ± 8.72 mU mg protein^-1^) (Figure 5) whereas *kivD* overexpression resulted in only partial complementation (19.61 ± 2.52 mU mg protein^-1^) (Figure 5). As *ARO10* overexpression could not restore ethanolic fermentation of Isoy21 and did not show any decarboxylation activity on pyruvate (data not shown), Aro10 was a promising candidate enzyme for increasing the decarboxylation of KIV for increased isobutanol production.

### Alcohol dehydrogenases

**Figure 6:**
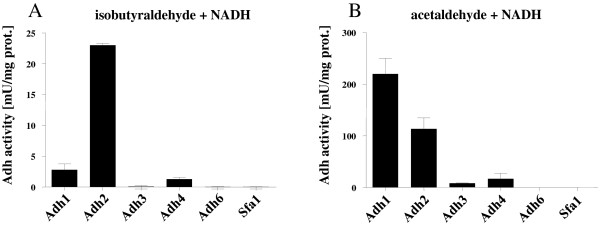
**ADH activities.** Strain JDY4 was transformed with plasmids overexpressing *ADH1*, *ADH2*, *ADH3*, *ADH4*, *ADH6* or *SFA1*. Transformants were grown in selective SCD medium and ADH activity with either 50 mM isobutyraldehyde (**A**) or 10 mM acetaldehyde (**B**) as a substrate determined in crude extracts and normalized to the amount of protein.

The conversion of isobutyraldehyde to isobutanol is the final enzymatic reaction in the isobutanol pathway. Enzymes which in principle might catalyze this reduction are encoded by the genes *ADH1ADH2ADH3ADH4ADH5* or *SFA1*, but the specificities of some of these enzymes to catalyze the reaction isobutyraldehyde to isobutanol remain unknown [19]. To determine the most suitable dehydrogenase for reduction of isobutyraldehyde, the *adh1 adh3 adh5* triple deletion strain JDY4 was transformed with plasmids overexpressing *ADH1ADH2ADH3ADH4ADH6* or *SFA1*. Both, isobutyraldehyde and acetaldehyde were tested as substrates (Figure 6). Whereas Adh1 overexpressing cells exhibited the highest activity with acetaldehyde (219.68 ± 21.3 mU mg protein^-1^), the highest activity with isobutyraldehyde was measured for Adh2 (22.98 ± 0.215 mU mg protein^-1^) (Figure 6). The overexpression of *ADH3ADH4ADH6* or *SFA1* resulted in no or only minor activities with both substrates. This experiment revealed Adh2 as a promising candidate enzyme for increased isobutyraldehyde reduction activity.

### Isobutanol fermentations with cells containing the cytosolic isobutanol pathway

In order to test whether the overexpression of the re-localized enzymes Ilv2ΔN54, Ilv5ΔN48 and Ilv3ΔN19 can increase production of isobutanol, fermentations in selective SCD medium without valine were performed under aerobic conditions in shake-flasks. Unexpectedly, CEN.PK2-1C containing empty vectors produced nearly the same amounts of isobutanol (13.70 ± 4.05 mg/L) as the strains overexpressing wild-type or truncated Ilv proteins (12.27 ± 0.90 mg/L and 10.31 ± 1.04 mg/L, respectively). Isobutanol production could not further be increased by the additional overexpression of *ARO10* and *ADH2* (not shown). Even when we used strain CEN.PK2-1C overexpressing the codon-optimized truncated *ILV* versions from plasmid p425-synthILV235, with or without simultaneous overexpression of *ARO10* and *ADH2*, we could not observe significant higher isobutanol production rates (not shown), isobutanol titers (up to 12.80 ± 2.44 mg/L and 11.96 ± 2.58 mg/L, respectively) and yields (0.30 ± 0.07 mg per gram of glucose and 0.27 ± 0.06 mg per gram of glucose, respectively).

We speculated that the lack of an increase in isobutanol production might be due to competition between the synthetic isobutanol pathway and the host valine biosynthesis pathway. Therefore, the triple *ilv* deletion strain Isoy17 (Δ*ilv2* Δ*ilv5* Δ*ilv3*) expressing the cytosolic isobutanol pathway was used for further fermentation experiments. Isoy17 was derived from Isoy16 by evolutionary engineering for improved growth in the absence of valine after expression of genes encoding truncated Ilv2ΔN54, Ilv5ΔN48 and Ilv3ΔN19, but was finally cured for the plasmids. In isobutanol fermentation experiments, however, it performed similar to Isoy16 (see below).

**Figure 7:**
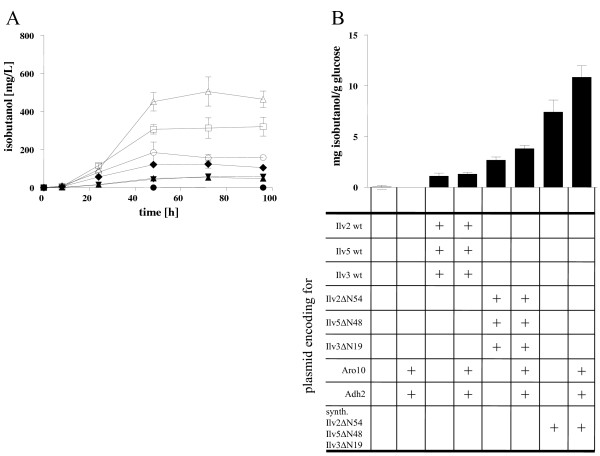
**Isobutanol production with strains expressing the cytosolic valine pathway.** Fermentation experiments were performed aerobically at 30°C in shake flasks in selective SCD-val medium containing 40 g/L glucose. Transformants were pregrown aerobically in fermentation medium, harvested and inoculated in fresh medium. Experiments were performed in triplicate with given standard deviations. Panel **A**: isobutanol production of Isoy17 expressing different plasmid combinations encoding for: empty vectors (closed circle); Aro10 and Adh2 (closed square); wild-type Ilv2, Ilv5 and Ilv3 (closed triangle), wild-type Ilv2, Ilv5, Ilv3, Aro10 and Adh2 (inverted closed triangle); truncated Ilv2ΔN54, Ilv5ΔN48 and Ilv3ΔN19 (closed diamond); truncated Ilv2ΔN54, Ilv5ΔN48, Ilv3ΔN19, Aro10 and Adh2 (open circle), codon-optimized truncated Ilv2ΔN54, Ilv5ΔN48 and Ilv3ΔN19 (open square), codon-optimized truncated Ilv2ΔN54, Ilv5ΔN48, Ilv3ΔN19, Aro10 and Adh2 (open triangle). Panel **B**: isobutanol yield of Isoy17 expressing different plasmid combinations encoding for genes which are indicated below the axis. Plus symbols denote overexpression of the indicated gene.

Isoy17 overexpressing wild-type *ILV* genes produced 52.57 ± 4.81 mg/L isobutanol within 96 hours (Figure 7). This was surprising as the corresponding wild-type strain overexpressing the same genes only produced 12.27 ± 0.90 mg/L (see above). Moreover, obviously this was not due to mutations selected in the evolutionary engineering optimization of Isoy17 from Isoy16, as in other isobutanol fermentations Isoy16 and 17 transformants performed nearly the same (see below). Additional overexpression of *ARO10* and *ADH2* increased isobutanol production only slightly (57.69 ± 3.87 mg/L) (Figure 7). In comparison, Isoy17 overexpressing the truncated *ILV* genes produced 123.77 ± 21.20 mg/L isobutanol. Moreover, additional overexpression of *ARO10* and *ADH2* resulted in a further increase up to 184.56 ± 55.00 mg/L with a yield of 3.81 ± 0.30 mg isobutanol per g of glucose (Figure 7). Glucose consumption rates of all the strains producing isobutanol were very similar and glucose was consumed after about 50-60 hours of fermentation (data not shown). These results show that overexpression of truncated *ILV* genes as compared to wild-type *ILV* genes resulted in a more than 2-fold increase in isobutanol production which could be even more increased by simultaneous overexpression of *ARO10* and *ADH2*. In comparison to the wild-type strain CEN.PK2-1C elimination of the competing mitochondrial valine biosynthesis pathway resulted in an about 13-fold increase in isobutanol production by the cytosolic isobutanol pathway.

### Isobutanol fermentations using codon-optimized *ILV* genes

As in the growth experiments in media lacking valine or isoleucine the codon-optimized *ILV* genes showed promising properties (Figure 4B), these genes were also tested in isobutanol fermentation experiments. For this purpose Isoy17 was transformed with the multicopy plasmid p425-synthILV235 expressing the three truncated *ILV* genes in a codon-optimized version behind strong promoters. For comparability with the former results, Isoy17 was additionally transformed with empty vectors eliminating the auxotrophic requirements of the cells. Plasmids overexpressing *ARO10* and *ADH2* were also transformed. After 96 hours of fermentation, Isoy17 containing p425-synthILV235 produced 320.40 ± 49.48 mg/L isobutanol with a yield of 7.41 ± 1.18 mg per g glucose (Figure 7). Additional overexpression of *ARO10* and *ADH2* increased isobutanol production up to 505.07 ± 76.29 mg/L with a yield of 10.85 ± 1.12 mg per gram of glucose (Figure 7). Again, glucose consumption rates of the strains producing isobutanol were very similar and glucose was consumed after about 50-60 hours of fermentation (data not shown). These results mean that yeast strains expressing the new codon-optimized cytosolic isobutanol pathway instead of the competing mitochondrial valine biosynthesis pathway produce more than 30-fold the amount of isobutanol.

### Isobutanol fermentations with cells containing single *ilv2,5* or *3* gene deletions

**Figure 8:**
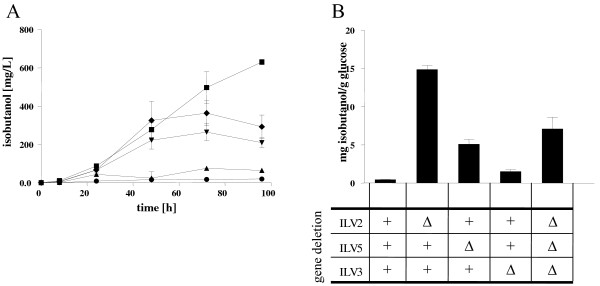
**Isobutanol production of different*ilv*single deletion strains expressing the codon-optimized cytosolic valine pathway.***S. cerevisiae* strains Isoy8 (Δ*ilv2*), Isoy10 (Δ*ilv3*), Isoy12 (Δ*ilv5*) and Isoy16 (Δ*ilv2*Δ*ilv5*Δ*ilv3*) were transformed with plasmid p425-synthILV235, pregrown and used for inoculation of fresh selective SCD-val containing 40 g/L glucose to an OD_600nm_ of 1. Fermentation experiments were performed aerobically in shake flasks at 30°C. The effect of different gene deletions were compared concerning productivity and yield of isobutanol in *S. cerevisiae* strains. CEN.PK2-1C transformed with p425-synthILV235 was used as a control. Panel **A**: isobutanol production of *S. cerevisiae* strains transformed with plasmid p425-synthILV235: CEN.PK2-1C (closed circle); Isoy8 (closed square); Isoy12 (closed inverted triangle); Isoy10 (closed triangle); Isoy16 (closed diamond). Panel **B**: isobutanol yield of different *ilv* deletion strains carrying p425-synthILV235. Deleted genes are indicated below the axis and are denoted with open triangle. Plus symbols denote intact genes.

The fermentation results with the triple *ilv* deletion strain indicate a competition between the mitochondrial valine and the cytosolic isobutanol pathway. In order to test whether the increase in isobutanol production was due to the deletion of the whole mitochondrial valine pathway or whether only the initial enzymatic reaction or one of the other two reactions is involved, fermentation experiments were performed with the *ilv* single deletion mutant strains Isoy8 (Δ*ilv2*), Isoy10 (Δ*ilv3*) and Isoy12 (Δ*ilv5*) transformed with p425-synthILV235, respectively. As controls, the wild-type strain CEN.PK2-1C and the non-evolved Isoy16 triple *ilv* strain were transformed with plasmid p425-synthILV235. As before, CEN.PK2-1C containing plasmid p425-synthILV235 exhibited very low isobutanol production (19.48 ± 0.99 mg/L) (Figure 8). Isoy16 containing p425-synthILV235 produced similar amounts of isobutanol (363.29 ± 65.77 mg/L) as was produced before by the evolved strain Isoy17 expressing p425-synthILV235. The *ilv2* single deletion strain where only the initial reaction of the mitochondrial valine pathway is blocked produced up to 630.27 ± 14.18 mg/L isobutanol with a yield of 14.86 ± 0.55 mg per g glucose (Figure 8). Isoy12 (Δ*ilv5* strain) expressing the codon-optimized cytosolic Ilv pathway produced 264.35 ± 44.96 mg/L and Isoy10 (Δ*ilv3*) produced 74.88 ± 7.53 mg/L isobutanol. These results indicate that the absence of an increase in isobutanol production in the wild-type can be explained by competition for pyruvate between the new cytosolic isobutanol pathway with the native mitochondrial pathway but that also the other enzyme reactions or metabolic intermediates of the mitochondrial valine pathway may interfere with the synthetic isobutanol production pathway.

## Discussion

The implementation of driving forces is important for high titer synthesis of biochemical products via genetic engineering (e.g. in [31]). Such driving forces might push or pull metabolic intermediates into, through or out of existing or engineered metabolic pathways. For the production of isobutanol with *S. cerevisiae* we have developed new driving forces which, when combined, resulted in the final production of more than 630 mg/L isobutanol with a yield of nearly 15 mg/g glucose. The highest values reported before for recombinant *S. cerevisiae* were about 150 mg/L isobutanol and a yield of 6.6 mg/g glucose [12,18].

Our strategy aimed to construct a cytosolic isobutanol production pathway. The driving force for this new pathway was provided by the simultaneous elimination of the competing mitochondrial valine synthesis pathway. This should increase the availability of intracellular pyruvate and should push pyruvate into the cytosolic isobutanol pathway. Overexpression of the cytosolically localized enzymes of valine biosynthesis in wild-type yeast cells did not increase isobutanol production but only when at least the first competing reaction of the mitochondrial pathway, Ilv2, was eliminated. Overexpression of the mitochondrially located wild-type enzymes did only slightly increase isobutanol production in *ilv* mutant cells but not in wild-type cells, in contrast to previous work [11]. These results might be explained either that the cytosolic KIV synthesis pathway is much more efficient than the mitochondrial pathway, that the transport of pyruvate into mitochondria or KIV out of mitochondria is limiting a mitochondrial pathway for isobutanol production and/or that the endogenous expression of wild-type Ilv enzymes (at least Ilv2) somehow has a negative effect on isobutanol production. To test the possibility of a simple competing role of the two pathways it will be highly revealing to delete the only very recently discovered genes coding for the mitochondrial pyruvate carrier [32].

As mitochondrial targeting sequences are not strictly defined we tested different versions of N-terminally truncated enzymes. Western blot and immunofluorescence analyses indicated for most of them that they were expressed and indeed located in the cytosol. To test enzymatic activities of the truncated versions, in the case of Ilv2 and Ilv3 we could use only a growth based assay as it was not possible to establish enzyme activity tests. When we expressed the two truncated Ilv2 versions in the cytosol but let Ilv5 and Ilv3 in the mitochondria we could detect slow growth on media lacking valine or isoleucine. This indicated that the enzymes were still able to convert pyruvate into 2-acetolactate which, however, only slowly crossed the mitochondrial membrane and could serve there as the substrate for Ilv5. In accordance with this, we also expressed the bacterial *ILV2* counterpart *alsS* from *B. subtilis* in the *ilv2* mutant strain which could complement the growth deficiency in the absence of valine comparable to Ilv2ΔN54 (not shown). Indeed, it is very likely that 2-acetolactate can cross the mitochondrial membrane as it is known that brewing yeasts produce diacetyl (2,3-butanedione) during fermentations. Production of diacetyl results from decarboxylation of 2-acetolactate outside the mitochondria [33,34]. In the case of Ilv5, nearly wild-type enzymatic activities for the re-located enzyme could be determined in enzyme assays. For the cytosolic Ilv3 versions Ilv3ΔN19 and Ilv3ΔN19^DE^ growth of *ilv3* mutants was completely restored in the absence of valine or isoleucine. As transport of KIV across the mitochondrial membrane is known [11] this indicates that DIV can be efficiently exported out of mitochondria. The three stronger truncated Ilv3 versions did not complement and probably have lost their enzymatic activities.

Normally, Ilv2 is regulated by Ilv6 [35]. As Ilv6 is involved in feedback inhibition of Ilv2 by branched-chain amino acids we omitted Ilv6 in the cytosolic isobutanol pathway. For *ilv5* mutants a petite phenotype is described as Ilv5 seems to be involved in the maintenance of wild-type mitochondrial DNA [36]. However, we could not find any indications for the occurrence of a petite phenotype in our *ilv5* mutant strains. Ilv3 is a [Fe-S] cluster containing enzyme [37]. In yeast cells, iron-sulphur clusters are normally synthesized within mitochdrondria by the ISC assembly machinery which is similar to the bacterial ISC system [38]. Extra mitochondrial iron-sulphur proteins are synthesized by the cytosolic assembly system CIA which requires both the mitochondrial ISC assembly and export machineries. Furthermore, for cytosolic and nuclear iron-sulphur protein biogenesis the CIA assembly machinery needs an unknown component which is exported by the ISC export machinery [39,40]. It seems that for the cytosolic Ilv3 version the loading with iron-sulphur can also be accomplished by the cytosolic assembly machinery CIA.

After we had successfully replaced the mitochondrial by a cytosolic valine pathway, we next optimized the flux through the new isobutanol pathway by adapting the codon usage of the valine biosynthesis genes to the codon usage of the highly expressed glycolytic genes of *S. cerevisiae*. The strongly expressed genes in *S. cerevisiae* like those coding for glycolytic proteins have adapted a highly biased codon usage with a strong preference for the most abundant tRNAs and can make up more than 50% of the proteins in a yeast cell. For most amino acids, the glycolytic genes are restricted to only one of the corresponding synonymous codons [22]. As we wanted to convert the anabolic valine pathway to a catabolic isobutanol pathway, we thought that it might be beneficial to adapt even the genes from the yeast valine pathway to the codon usage of the catabolic glycolytic pathway in yeast. Thereby the codon adaptation index (CAI) values of the three valine synthesis enzymes were changed from 0.356 (*ILV2ΔN54*), 0.448 (*ILV3ΔN19*) and 0.846 (*ILV5ΔN48*) to 0.991 (*ILV2ΔN54*), 0.987 (*ILV3ΔN19*) and 0.992 (*ILV5ΔN48*), respectively. The CAI measures the deviation of a given protein coding gene sequence with respect to a reference set of genes which here are the highly expressed yeast genes [41]. Indeed, by turning the ‘anabolic’ genes into highly expressed 'catabolic' genes, we could significantly improve production of isobutanol. This demonstrates that even expression of endogenous yeast genes can be increased by converting them into the glycolytic codon usage.

The next crucial driving force was the valine requirement of our yeast strains. As KIV is not only the final substrate for the transamination reaction into valine but also an intermediate in the isobutanol production pathway, we argued that the valine requirement would serve as a pulling force to increase the production of KIV. Indeed, only in the absence of valine we could observe significant isobutanol production in the triple *ilv* mutant strain expressing the truncated Ilv enzymes. When we added valine to the fermentation medium only basal levels of isobutanol production could be observed.

To complete the isobutanol pathway a suitable KIV decarboxylase (KDC) and an alcohol dehydrogenase with high activity on isobutyraldehyd had to be found. Indeed, overexpression of *ARO10* restored KIV decarboxylase activity in a *pdc1, 5, 6* mutant strain but not pyruvate decarboxylase activity. Moreover, already in a previous work enhanced Aro10 activity had resulted in increased isobutanol production [12,18]. As also the bacterial homologue KivD of *L. lactis* had been used successfully in other studies [7,18], we included it in the enzymatic assays. However, as our construct performed worse than Aro10, we did not test it in fermentations. The last enzymatic reaction of the isobutanol pathway can be catalyzed by different yeast oxidoreductases [19,42]. To find an enzyme with a high activity on isobutyraldehyde we overexpressed *ADH1ADH2ADH3ADH4ADH6* and *SFA1* and tested them by *in vitro* enzyme assays. Whereas Adh1 had the highest activity with acetaldehyde, Adh2 had the highest activity with isobutyraldehyd and NADH. Moreover, in previous work overexpression of *ADH2* was successfully used for isobutanol production [7,9,43]. Indeed, by overexpressing *ADH2* simultaneously with *ARO10* we could further increase isobutanol yields. We could not confirm high activity of Adh6 on isobutyraldehyde as had been suggested previously [12].

It should be stressed that the isobutanol concentrations reported in our study are clearly underestimated. In a control experiment we observed that 35% of the isobutanol was lost during 5 days of incubation at 30°C due to evaporation. Moreover, in our experiments isobutanol production stopped when glucose was exhausted. Therefore, feeding more glucose would certainly increase isobutanol titers. Nevertheless, the most promising step to push more pyruvate into the new pathway is to delete pyruvate decarboxylase and to replace ethanol fermentation completely by isobutanol fermentation. This, however, would create new problems as *pdc*^*-*^ mutants are known to become auxotrophic for cytosolic acetyl-CoA which is needed for e.g. lipid synthesis [44]. Moreover, whereas in glycolysis NADH is produced as a reduced cofactor, Ilv5 in the isobutanol pathway exclusively uses NADPH. As *S. cerevisiae* lacks transhydrogenases to transfer hydride-ions from NADH to NADP^+^, the cofactor levels within the cell will be imbalanced [45]. Therefore, either the yeast glycolytic glyceraldehydes dehydrogenase must be exchanged against an NADP^+^ dependent enzyme (together with alcohol dehydrogenase) or the cofactor preference of Ilv5 must be changed to NADH. Such a strategy could be successfully established in *E. coli* where under anaerobic conditions isobutanol production reached nearly 100% of the theoretical yield [46]. The final challenge, however, would be to overcome the toxicity of isobutanol on microbial organisms. For this, the most promising way is to find effective methods to extract isobutanol already during the fermentations.

## Conclusions

In this work, we expressed a valine biosynthetic pathway from pyruvate to KIV in the cytosol of yeast cells. Simultaneous blocking of the mitochondrial pathway and omission of valine from the fermentation medium pushed and pulled pyruvate into and through the new pathway. Changing the ‘anabolic’ codon usage of valine synthesis genes into a ‘catabolic’ codon usage further improved flux through the new pathway. Overexpression of KDC and ADH activities increased the conversion of KIV to isobutanol. The highest measured isobutanol titer of 0.6 g/L represents the highest titer ever reported for recombinant *S. cerevisiae*.

## Methods

### Strains and media

Yeast strains used in this work are listed in Table 1 and plasmids in Table S1 (see Additional file 1: Table S1) [47-50]. *S. cerevisia* was grown in selective medium (1.7 g/L Difco yeast nitrogen base without amino acids and 5 g/L ammonium sulfate), supplemented with amino acids but omitting the selective plasmid markers nutrients as described previously [51], containing 2% glucose as sole carbon source (SCD). Compared to SCD medium, SMD medium was synthetic medium only supplemented for auxotrophic requirements. For maintenance of resistance plasmids, media contained appropriate concentration of antibiotics. Concentrations for geneticin were 200 mg/L, for hygromycin B 200 mg/L and 100 mg/L for nourseothricin. “Selective medium” means medium without auxotrophic requirements or with antibiotics for plasmid selection.

**Table 1:** ***S. cerevisiae*** **strains used in this work**

**Strains**	**Relevant genotype**	**Source**
CEN.PK2-1C	*MAT*a *leu2-3,112 ura3-52 trp1-289 his3*-_*1 MAL2-8c SUC2*	EUROSCARF, Frankfurt
Isoy8	*MAT*a *leu2-3,112 ura3-52 trp1-289 his3*-_*1 MAL2-8c SUC2* Δ*ilv2::loxP*	This work
Isoy10	*MAT*a *leu2-3,112 ura3-52 trp1-289 his3*-_*1 MAL2-8c SUC2* Δ*ilv3::loxP*	This work
Isoy12	*MAT*a *leu2-3,112 ura3-52 trp1-289 his3*-_*1 MAL2-8c SUC2* Δ*ilv5::loxP*	This work
Isoy16	*MAT*a *leu2-3,112 ura3-52 trp1-289 his3*-_*1 MAL2-8c SUC2* Δ*ilv2::loxP* Δ*ilv5::loxP* Δ*ilv3::loxP*	This work
Isoy17	*MAT*a *leu2-3,112 ura3-52 trp1-289 his3*-_*1 MAL2-8c SUC2* Δ*ilv2::loxP* Δ*ilv5::loxP* Δ*ilv3::loxP*; unknown beneficial mutations for growth on media lacking valine	This work
Isoy21	*MAT*a *leu2-3,112 ura3-52 trp1-289 his3*-_*1 MAL2-8c SUC2* Δ*pdc1::loxP* Δ*pdc5::loxP* Δ*pdc6::loxP*; unknown beneficial mutations for growth on media with glucose as sole carbon source	This work
JDY4	*MAT*a *leu2-3,112 ura3-52 trp1-289 his3*-_*1 MAL2-8c SUC2* Δ*adh1::loxP* Δ*adh3::loxP* Δ*adh5::loxP-kanMX-loxP*	Boles, lab stock

For serial dilution growth assays using single Δ*ilv* deletion strains, cells expressing *ILV2*, *ILV3* or *ILV5* variants were cultivated till exponential phase in selective SCD media. Cells were collected and resuspended in sterile water to an OD_600nm_ of 1. Cultures were serially diluted in 10-fold steps and 7 μl of each dilution was spotted on selective SMD agar plates. As a positive control, selective SMD media were supplemented with leucine, isoleucine and valine. To investigate valine and isoleucine requirements, transformants were also spotted on SMD + leu media lacking valine or isoleucine or both. Plates were incubated at 30°C up to one week. For growth assays with ilv2 and ilv5 mutants 0.5% ammonium sulfate was used as nitrogen source whereas 0.5% leucine + 0.5% isoleucine + 0.5% valine was employed as nitrogen source for ilv3 mutants. For serial dilution growth assays using triple Δ*ilv* deletion strains, tranformants containing plasmids encoding for different combinations of Ilv2, Ilv5 and Ilv3 variants were streaked out or replica plated onto selective SMD + leu + ile media lacking valine. After colonies appeared they were collected and resuspended in sterile water to an OD_600nm_ of 1. Cultures were serially diluted in 10-fold steps and 7 μl of each dilution was spotted on selective SMD + leu agar plates containing valine and/or isoleucine, with 0.5% proline as sole nitrogen source. In aerobic batch cultivations, *S. cerevisiae* was grown in selective SCD media.

Plasmids were amplified in *Escherichia coli* strain DH5α (Gibco BRL, Gaithersburg, MD) and strain SURE (Strata gene, La Jolla, CA). *E. coli* transformations were performed via electroporation according to the methods of Dower et al., (1988) [52]. *E. coli* was grown on LB (Luria-Bertani) medium with 40 μg/ml ampicillin for plasmid selection.

### Construction of *ilv* deletion strains

Strains Isoy8, Isoy10, Isoy12 and Isoy16 were constructed employing the *lox::kanMX::loxP*/Cre recombines system and the 'short flanking homology PCR' technology [47]. Instead of geneticin resistance (*kanMX*) gene, hygromycine B resistance gene (*hphNT1*) and nourseothricin resistance gene (*natNT2*) were also used to generate *ilv* deletion mutants. The primers used for the construction of the replacement PCR constructs are listed (see Additional file 1: Table S2). Primers were obtained from biomers.net. Yeast transformations were carried out as described previously [53,54]. As induction of the galactose-inducible, glucose-repressible Cre recombines on plasmid pSH47 by galactose appeared to have deleterious effects on cells containing several *loxP*-sites, we routinely used maltose (which has a weaker repressive effect than glucose) to induce/derepress *loxP*-Cre recombination.

### Plasmid construction

Plasmids and primers used in this publication are listed in Table S1 and Table S2, respectively (see Additional file 1). The coding regions of *ILV2, ILV5, ILV3, ARO10, ADH1,ADH2, ADH3, ADH4, ADH6* and *SFA1* of *S. cerevisiae* strain CEN.PK2-1C were amplified by PCR, respectively, and cloned into the linearized vectors p423H7, p424H7, p426H7, pRS42KH7, pRS42HH7 and pRS42NH7 by recombination cloning omitting the six histidine codons [55].

Furthermore, ORFs of *ILV2, ILV5, ILV3* and truncated variants of *ILV2*, *ILV5* and *ILV3* of CEN.PK2-1C were also cloned by recombination cloning into the vector p423H7, p426H7 and pRS42KH7, respectively, fusing six histidine codons at their 3’-terminal ends. In addition, the ORF of *kivD* of *Lactococcus lactis* was fused with six histidine codons at the 5'-terminal end.

Codon-optimized ORF versions of *ILV2ILV5* and *ILV3* were obtained from Geneart AG (Regensburg, Germany) by changing the original codons of the respective genes to those used by the genes encoding glycolytic enzymes in *S. cerevisiae*[22]. The codon-optimized *ILV2ΔN54* was cloned behind a truncated *HXT7* promoter fragment [56] and *CYC1* terminator, the codon-optimized *ILV5ΔN48* behind *FBA1* promoter and *PGK1* terminator and the codon-optimized *ILV3ΔN19* was under control of *PFK1* promoter and *FBA1* terminator. Furthermore, the plasmid contained two nucleotide sequences (369 bp and 385 bp) homologous to the yeast intergenic *FMO1*-locus, which could be useful for integration into chromosomVIII. As selection marker, it contained the geneticin resistance gene (*kanMX*) flanked by *loxP-*sites. Codon-optimized truncated *ILV*-ORFs, promoters and terminators were amplified with primers listed (see Additional file 1: Table S2). Linearized vector p425H7, amplified codon-optimized truncated *ILV* ORFs, amplified promoter/terminator elements (see Additional file 1 for plasmid p425-synthILV235), amplified *loxP-kanMX-loxP* resistance gene and 369 bp and 385 bp homologous to *FMO1*-locus were transformed into Isoy16. Transformants were replica plated on selective media lacking valine to select clones containing functional vectors which enabled to complement valine auxotrophy.

Molecular techniques were performed according to published procedures [57]. Yeast transformations and resolution of plasmid DNA from yeast cells were carried out as described previously [53,58].

### Metabolite analysis

The concentrations of glucose, ethanol, glycerol and acetate were determined by high-performance liquid chromatography (Dionex) using a Nugleogel Sugar 810 H exchange column (Macherey-Nagel GmbH & Co, Germany). The column was eluted with 5 mM H_2_SO_4_ as mobile phase and a flow rate of 0.6 ml/min at the temperature of 65°C. Detection was done by means of a Shodex RI-101 refractive index detector. For data evaluation, Chromeleon software (version 6.50) was used. Rates of glucose consumption were determined in the phase of glucose growth.

Isobutanol concentration was measured by using static head-space-gas chromatography combined with mass spectrometry. The gas chromatograph (model 7890A, Agilent, Waldbronn, Germany) was equipped with a CTC PAL Combi XT auto sampler (CTC Analytics AG, Zwingen, Switzerland) and a Series 5975C (Agilent, Waldbronn, Germany) mass selective detector. Analyses were separated on a DB5ht column (lenght of 30 m, 0.25 mm of an inner diameter, 0,1 μm in strength of stationary phase film; Agilent, Waldbronn, Germany). Helium was used as the carrier gas at a constant flow rate of 1 ml/min. Samples (2 ml in 20 ml sealed head space vials) were investigated by applying the headspace option. After incubation in the sample oven for five minutes at 95°C 800 μl of the gas phase were aspirated and injected into the gas chromatograph. The method parameters were as follows: inlet temperature: 250°C; injection mode: Split, ratio 10:1; oven temperature program: 35°C for 1 min, increased to 50°C with 10°C/min and finally to 200°C with 120°C/min, hold for 3 min before re-equilibration. The temperature of the transfer line to the mass selective detector was held at 280°C, the ion sources temperature 230°C and its quadrupole temperature at 150°C. Mass data were recorded with a Scan/SIM combination of 45-100D and 74.1D, respectively. For data evaluation and quantification the Data Analysis tool from MSD ChemStation E.02.00.493 (Agilent, Waldbronn, Germany) was used. The single ion chromatogram of 74.1D, which corresponds to the molecular ion species of isobutanol was integrated and isobutanol concentration in per cent by volume were inferred from a calibration line.

### Batch fermentations

Cultures of laboratory strains (100 ml) were grown in 500-ml shake flasks at 30°C with constant shaking at 180 rpm. Precultures were grown in selective SCD medium containing 4% glucose as the sole carbon source. Cells were washed with sterile water and inoculated to an optical density at 600 nm (OD_600nm_) of 1 in the same medium. During the fermentations equivalent volumes of fermentation medium were added to cultures after taking samples for metabolite and isobutanol analysis in order to compensate for volume losses. The dilutions were considered in the calculations of metabolite concentrations. Fermentations were started with different precultures and were performed in triplicate with the given standard deviations.

### Western blot analysis

To test whether the N-terminally truncated Ilv enzymes were expressed and were not degraded as a result of their truncation and re-localization Western blot analyses were performed. The plasmids expressing the wild-type and truncated ORFs with 6His-tags at their C-termini were transformed into strain CEN.PK2-1C. Transformants were grown on selective SCD media into the exponential growth phase. Cells were harvested and disrupted with Y-PER®, which was used as recommended from provider (Thermo Scientific). The protein content was determined according to the method of Bradford (1976) and adjusted for equal loading on a sodium dodecyl sulfate (SDS)-polyacrylamide gel [59]. Twenty micrograms of total protein was applied in each lane. Preparation of cells was also performed as described in Kushnirov (2000) [60]. For Western blot analysis, protein was transferred from the SDS gel to PVDF membranes by submerse electro blotting. Ilv-6His were detected with mouse anti-His6 antibody (Roche) and goat anti-mouse immunoglobulin G coupled to peroxidase (Roche).

### Subcellular localization with indirect immunofluorescence microscopy

To localize truncated ILV enzymes in yeast indirect immunofluorescence microscopy was performed using CLSM (Confocal Laser Scanning Microscopy; TCS SP5 Leica Microsystems AG, Wetzlar, Germany). Therefore, yeast transformants expressing C-terminally His6 epitope-tagged variants of *ILV2, ILV5* and *ILV3* from *S. cerevisiae* (carried on multicopy vectors) were cultivated until early exponential growth phase in selective SCD medium. An appropriate volume of cells was treated with 1/3 volume of PFA/PBS. Cells were washed two times with TDES buffer (100 mM Tris–HCl pH 7.5; 5 mM EDTA; 25 mM DTT; 1.2 M Sorbitol) and one time with 0.2 M phosphate/citrate buffer. After centrifugation the cell pellet was incubated for one hour at 30°C in 0.2 M phosphate/citrate buffer containing zymolyase (1 mg/ml). After incubation cells were washed two times with PBS. Cell pellet was resuspended in 0.5% Triton X 100/PBS and incubated for 10 min at RT. Spheroplasts were immobilized on coated cover slips which were treated before using with poly-L-lysine. After incubation of 10 min slips were washed two times with PBS and treated with 100 mM Glycin/PBS for 15 min to block residual aldehyde groups. Following, slips were blocked over night with 5% BSA/PBS. Blocked slips were treated with primary antiserum in an appropriate dilution in 1-5% BSA/PBS for at least 3 h followed by two washing steps in PBS. For C-terminally His6 epitope-tagged variants of *ILV2, ILV5* and *ILV3* variants mouse anti-His6 and as a control rabbit anti-Hsp70 were used. After washing steps slips were incubated with secondary fluorochrome-labeled antiserum (anti-mouse-cy3 1:500) in an appropriate dilution in 5% BSA/PBS for 2 h. Immobilized cells were washed one time with PBS and conserved in Aqua Poly/Mount (Polysciences, Inc.). To detect enzymes with bounded antibodies followed secondary antiserum were used, donkey anti-mouse immunoglobulin G coupled to Cy3 for C-terminally His6 epitope-tagged enzymes and goat anti-rabbit immunoglobulin G coupled to Cy2 for Hsp70 SSB1.

Stacks of images were restored using *Huygens.* Location of enzymes in prepared cells were evaluated by using Imaris 4.1.3 software (Bitplane AG, Zurich, Switzerland) and Photoshop CS 2 software (Adobe Systems, San Jose, USA).

### Enzyme assays

To measure enzyme activities, yeast transformants were cultivated until early exponential growth phase in selective SCD medium. Cells were harvested and disrupted with glass beads (diameter, 0.45 mm) using a Vibrax cell disrupter (Janke & Kunkel, Staufen, Germany). Protein concentration was determined with the method of Bradford (1976) by using bovine serum albumin as a standard [59]. Enzyme assays were performed immediately after preparation of crude extracts. One unit of enzyme activity was defined as conversion of one μmol substrate per minute.

#### Ilv5 assay

To confirm enzyme activity of truncated Ilv5, Isoy12 expressing truncated *ILV5ΔN48* (carried on multicopy vector) was investigated. As a control Isoy12 was transformed with an empty vector or vector encoding wild-typ Ilv5. Assays were carried out in reaction mixtures containing 0.23 mM NADPH, 2 mM MgCl_2_ in 50 mM Tris–HCl buffer (pH 7.4), and crude cell extracts. The reaction was started by addition of 3-4 mM acetolactate and monitored by measuring oxidation of NADPH spectrophotometrically at 340 nm. All enzyme assays were carried out at least in triplicate. Synthesis of acetolactate was based on Krampitz (1948) and production was confirmed through NMR spectroscopic analysis [61]. 1 H – Spectra were made on a 400 MHz spectrometer (Bruker BioSpin GmbH, Germany) with 200 scans and spectral width of 7. As reference the water signal was set at 4.7 ppm as it is in accordance with the signal range for water protons. For the deuteriumlock 10% deuteriumoxid was admixed to the aqueous solution. The puls sequence was a 90° puls. Following, the FID (Free Induction Decay) was recorded and converted by Fourier Transformation in absorptive signals. Prepared spectra were evaluated by using TopSpin software (Bruker, Germany).

#### Aro10 assay

KIV decarboxylase activity in cell extracts of recombinant yeast strains was determined at 30°C. Strain Isoy21 was transformed with p424H7-Aro10 and p424H7-kivD, respectively, to investigate enzyme activity, and as a control Isoy21 and CEN.PK2-1C containing empty vector were used, respectively. Assays were carried out in reaction mixtures containing 0.23 mM NADH, 2 U alcohol dehydrogenase in 40 mM imidazolbuffer (buffer contained 40 mM imidazol, 5 mM MgCl_2_, 0.2 mM thiaminepyrophosphate, pH 7.0 was adjusted with KOH), and crude cell extracts, as described previously [44]. The reaction was started by addition of 2 mM KIV and monitored by measuring oxidation of NADH spectrophotometrically at 340 nm. *Pdc1,5,6* triple mutants expressed only a very low KDC activity (8.66 ± 0.41 mU mg protein^-1^) whereas the wild-type strain exhibited the highest activity (38.73 ± 6.24 mU mg protein^-1^).

#### Adh assay

The enzymes Adh1, Adh2, Adh3, Adh4, Adh6 and Sfa1 were investigated to determine the specific enzyme activity towards isobutyraldehyde and acetaldehyde in crude cell extracts. The plasmids encoding these enzymes were transformed into JDY4. Assays were carried out in reaction mixtures containing 0.23 mM NADH in 50 mM MOPS (pH 7), and crude cells extracts. The reaction was started by addition of substrates isobutyraldehyde (50 mM) or acetaldehyde (10 mM). The oxidation of NADH was monitored spectrophotometrically at 340 nm.

## Abbreviations

ADH, Alcohol dehydrogenase; ALAC, 2-acetolactate; amino acid D, Aspartic acid; amino acid E, Glutamic acid; CAI, Codon adaptation index; CIA, Cytosolic iron-sulfur cluster assembly; DIV, 2,3-dihydroxyisovalerate; FID, Free induction decay; GAP, Glyceraldehyde-3-phosphate; IBA, Isobutyraldehyde; ISC, Iron-sulfur cluster; KDC, Ketoacid decarboxylase; KIV, 2-ketoisovalerate; MTS, Mitochondrial targeting sequence; OD600nm, Optical density at 600 nm; ORF, Open reading frame; PYR, Pyruvate; SCD, Synthetic complete medium containing glucose; SMD, Synthetic minimal medium containing glucose; 2 U, 2 units.

## Competing interests

The authors declare competing financial interests. EB is co-founder and shareholder of the Swiss biotech company Butalco GmbH.

## Authors’ contributions

DB designed and performed most of the experiments and wrote the first draft of the manuscript. CW contributed to the design of the work and performed some of the experiments. WL and HBB established GC/MS methods, and WL and DB performed GC/MS analyses. EB initiated this work, developed the experimental design and edited the final manuscript. All authors read and approved the final manuscript.

## Supplementary Material

Additional file 1 **Table S1.** Plasmids used in this work; **Table S2.** Oligonucleotides used in this work. Click here for file
