# Supplementary material for: Cytosolic re-localization and optimization of valine synthesis and catabolism enables inseased isobutanol production with the yeast Saccharomyces cerevisiae
Source: Biotechnol Biofuels. 2012 Sep 6;5:65. doi: 10.1186/1754-6834-5-65 (PMC3476451; doi:10.1186/1754-6834-5-65)
Supplement: Additional file 1 — Table S1. Plasmids used in this work; Table S2. Oligonucleotides used in this work. [file 1754-6834-5-65-S1.docx]

**Additional file1**

**Table 2** Plasmids used in this work.

| Plasmid | Relevant characteristics | Source or reference |
| --- | --- | --- |
| pUG6 | DNA-template for amplification of *loxP-kanMX-loxP* gene resistance marker gene | [49] |
| pCZ1 | DNA-template for amplification of *loxP-hphNT1-loxP* gene resistance marker gene | [59] |
| pCZ2 | DNA-template for amplification of *loxP-natNT2-loxP* gene resistance marker gene | [59] |
| pSH47 | Cre-recombinase under control of *GAL1* promoter; *URA3* marker gene | [49] |
| p423H7 | 2μ-plasmid with shortened *HXT7* promoter and *CYC1* terminator; *HIS3* marker gene | [60] |
| p424H7 | 2μ-plasmid with shortened *HXT7* promoter and *CYC1* terminator; *TRP1* marker gene | [60] |
| p426H7 | 2μ-plasmid with shortened *HXT7* promoter and *CYC1* terminator; *URA3* marker gene | [60] |
| pRS42K | 2μ-plasmid with shortened *HXT7* promoter and *CYC1* terminator; *kanMX* marker gene | [61] |
| pRS42N | 2μ-plasmid with shortened *HXT7* promoter and *CYC1* terminator; *natNT2* marker gene | [61] |
| pRS42KH7 | 2μ-plasmid with shortened *HXT7* promoter and *CYC1* terminator; *kanMX* marker gene | This work |
| pRS42HH7 | 2μ-plasmid with shortened *HXT7* promoter and *FBA1* terminator; *hphNT1* marker gene | This work |
| pRS42NH7 | 2μ-plasmid with shortened *HXT7* promoter and *CYC1* terminator; *natNT2* marker gene | This work |
| p424H7-Aro10 | 2μ-plasmid; *ARO10* of *S.* *cerevisiae* under control of shortened *HXT7* promoter and *CYC1* terminator, *TRP1* marker gene | This work |
| p424H7-kivD | 2μ-plasmid expressed with a N-terminal, 6-fold Histidin tagged, version of *kivD* of *Lactococcus lactis* under control of shortened *HXT7* promoter and *CYC1* terminator, *TRP1* marker gene | This work |
| p426H7-Adh1 | 2μ-plasmid; *ADH1* of *S.* *cerevisiae* under control of shortened *HXT7* promoter and *CYC1* terminator, *URA3* marker gene | This work |
| p426H7-Adh2 | 2μ-plasmid; *ADH2* of *S.* *cerevisiae* under control of shortened *HXT7* promoter and *CYC1* terminator, *URA3* marker gene | This work |
| p426H7-Adh3 | 2μ-plasmid; *ADH3* of *S.* *cerevisiae* under control of shortened *HXT7* promoter and *CYC1* terminator, *URA3* marker gene | This work |
| p426H7-Adh4 | 2μ-plasmid; *ADH4* of *S.* *cerevisiae* under control of shortened *HXT7* promoter and *CYC1* terminator, *URA3* marker gene | This work |
| p426H7-Adh6 | 2μ-plasmid; *ADH6* of *S.* *cerevisiae* under control of shortened *HXT7* promoter and *CYC1* terminator, *URA3* marker gene | This work |
| p426H7-Sfa1 | 2μ-plasmid; *SFA1* of *S.* *cerevisiae* under control of shortened *HXT7* promoter and *CYC1* terminator, *URA3* marker gene | This work |
| p426H7-ILV2∆N54tag | 2μ-plasmid expressed with a C-terminal, 6-fold Histidin-tagged, N-terminal truncated version of 54 amino acids of *S. cerevisiae* *ILV2* under control of shortened *HXT7* promoter and *CYC1* terminator, *URA3* marker gene | This work |
| p426H7-ILV2∆N85tag | 2μ-plasmid expressed with a C-terminal, 6-fold Histidin-tagged, N-terminal truncated version of 85 amino acids of *S. cerevisiae* *ILV2* under control of shortened *HXT7* promoter and *CYC1* terminator, *URA3* marker gene | This work |
| p426H7-ILV2wttag | 2μ-plasmid expressed with a C-terminal, 6-fold Histidin-tagged, full length version of *S. cerevisiae* *ILV2* under control of shortened *HXT7* promoter and *CYC1* terminator, *URA3* marker gene | This work |
| pRS42KH7-ILV3∆N19tag | 2μ-plasmid expressed with a C-terminal, 6-fold Histidin-tagged, N-terminal truncated version of 19 amino acids of *S. cerevisiae* *ILV3* under control of shortened *HXT7* promoter and *CYC1* terminator, *kanMX* marker gene | This work |
| pRS42KH7-ILV3∆N34tag | 2μ-plasmid expressed with a C-terminal, 6-fold Histidin-tagged, N-terminal truncated version of 34 amino acids of *S. cerevisiae* *ILV3* under control of shortened *HXT7* promoter and *CYC1* terminator, *kanMX* marker gene | This work |
| pRS42KH7-ILV3∆N42tag | 2μ-plasmid expressed with a C-terminal, 6-fold Histidin-tagged, N-terminal truncated version of 42 amino acids of *S. cerevisiae* *ILV3* under control of shortened *HXT7* promoter and *CYC1* terminator, *kanMX* marker gene | This work |
| pRS42KH7-ILV3∆N50tag | 2μ-plasmid expressed with a C-terminal, 6-fold Histidin-tagged, N-terminal truncated version of 50 amino acids of *S. cerevisiae* *ILV3* under control of shortened *HXT7* promoter and *CYC1* terminator, *kanMX* marker gene | This work |
| pRS42KH7-ILV3∆N19DEtag | 2μ-plasmid expressed with a C-terminal, 6-fold Histidin-tagged, N-terminal truncated version of 19 amino acids of *S. cerevisiae* *ILV3* with an additional insertion of aspartate and glutamate under control of shortened *HXT7* promoter and *CYC1* terminator, *kanMX* marker gene | This work |
| pRS42KH7-ILV3wttag | 2μ-plasmid expressed with a C-terminal, 6-fold Histidin-tagged, full length version of *S. cerevisiae* *ILV3* under control of shortened *HXT7* promoter and *CYC1* terminator, *kanMX* marker gene | This work |
| p426H7-ILV5∆N48tag | 2μ-plasmid expressed with a C-terminal, 6-fold Histidin-tagged, N-terminal truncated version of 48 amino acids of *S. cerevisiae* *ILV5* under control of shortened *HXT7* promoter and *CYC1* terminator, *URA3* marker gene | This work |
| p426H7-ILV5wttag | 2μ-plasmid expressed with a C-terminal, 6-fold Histidin-tagged, full length version of *S. cerevisiae* *ILV5* under control of shortened *HXT7* promoter and *CYC1* terminator, *URA3* marker gene | This work |
| p426H7-ILV2∆N54 | 2μ-plasmid; truncated version of *S. cerevisiae* *ILV2* lacking N-terminal 54 amino acids under control of shortened *HXT7* promoter and *CYC1* terminator, *URA3* marker gene | This work |
| p426H7-ILV2∆N85 | 2μ-plasmid; truncated version of *S. cerevisiae* *ILV2* lacking N-terminal 85 amino acids under control of shortened *HXT7* promoter and *CYC1* terminator, *URA3* marker gene | This work |
| p426H7-ILV2wt | 2μ-plasmid; *ILV2* of *S.* *cerevisiae* under control of shortened *HXT7* promoter and *CYC1* terminator, *URA3* marker gene | This work |
| pRS42KH7-ILV3∆N19 | 2μ-plasmid; truncated version of *S. cerevisiae* *ILV3* lacking N-terminal 19 amino acids under control of shortened *HXT7* promoter and *CYC1* terminator, *kanMX* marker gene | This work |
| pRS42KH7-ILV3∆N34 | 2μ-plasmid; truncated version of *S. cerevisiae* *ILV3* lacking N-terminal 34 amino acids under control of shortened *HXT7* promoter and *CYC1* terminator, *kanMX* marker gene | This work |
| pRS42KH7-ILV3∆N42 | 2μ-plasmid; truncated version of *S. cerevisiae* *ILV3* lacking N-terminal 42 amino acids under control of shortened *HXT7* promoter and *CYC1* terminator, *kanMX* marker gene | This work |
| pRS42KH7-ILV3∆N50 | 2μ-plasmid; truncated version of *S. cerevisiae* *ILV3* lacking N-terminal 50 amino acids under control of shortened *HXT7* promoter and *CYC1* terminator, *kanMX* marker gene | This work |
| pRS42KH7-ILV3∆N19DE | 2μ-plasmid; truncated version of *S. cerevisiae* *ILV3* lacking N-terminal 19 amino acids with inserted glutamate and aspartate under control of shortened *HXT7* promoter and *CYC1* terminator, *kanMX* marker gene | This work |
| pRS42KH7-ILV3wt | 2μ-plasmid; *ILV3* of *S.* *cerevisiae* under control of shortened *HXT7* promoter and *CYC1* terminator, *kanMX* marker gene | This work |
| p423H7-ILV5∆N48 | 2μ-plasmid; truncated version of *S. cerevisiae* *ILV5* lacking N-terminal 48 amino acids under control of shortened *HXT7* promoter and *CYC1* terminator, *HIS3* marker gene | This work |
| p423H7-ILV5wt | 2μ-plasmid; *ILV5* of *S.* *cerevisiae* under control of shortened *HXT7* promoter and *CYC1* terminator, *HIS3* marker gene | This work |
| pRS42NH7-ARO10 | 2μ-plasmid; *S. cerevisiae* *ARO10* under control of shortened *HXT7* promoter and *CYC1* terminator, *natNT2* marker gene | This work |
| pRS42HH7-ADH2 | 2μ-plasmid; *S. cerevisiae* *ARO10* under control of shortened *HXT7* promoter and *FBA1* terminator, *hphNT1* marker gene | This work |
| p425-synthILV235 | 2μ-plasmid with integrative ILV-cassette which contains truncated ORFs of codon-optimized *ILV2∆N54*, codon-optimized *ILV5∆N48* and codon-optimized *ILV3∆N19* of *S. cerevisiae*; codon-optimized *ILV2∆N54* under control of shortened *HXT7* promoter and *CYC1* terminator, codon-optimized *ILV5∆N48* under control of *FBA1* promoter and *PGK1* terminator, codon-optimized *ILV3∆N19* under control of *PFK1* promoter and *FBA1* terminator, *loxP*-*kanMX*-*loxP* resistance gene, flanked of 369bp and 385bp homologous to *FMO1*-locus, respectively, *LEU2* marker gene in 2μ-plasmid; capability of integration into chromosomVIII of codon-optimized ILV-cassette through *in vivo*-recombination after restriction by *Asc*I/*Pac*I | This work |

**Table 3** Oligonucleotides used in this work.

| Oligonucleotides | Sequence | Used for construction of |
| --- | --- | --- |
| Ilv2-wt-f | AACACAAAAACAAAAAGTTTTTTTAATTTTAATCAAAAAATGATCAGACAATCTACGCTAA | p426H7-ILV2wt, p426H7-ILV2wttag |
| ILV2∆N54-f | AACACAAAAACAAAAAGTTTTTTTAATTTTAATCAAAAAATGCCAGAGCCTGCTCCAAG | p426H7-ILV2∆N54, p426H7-ILV2∆N54tag |
| ILV2∆N85-f | AACACAAAAACAAAAAGTTTTTTTAATTTTAATCAAAAAATGGATACCTCTTTCGTCGG | p426H7-ILV2∆N85, p426H7-ILV2∆N85tag |
| ILV2-r | GAATGTAAGCGTGACATAACTAATTACATGACTCGAGTCAGTGCTTACCGCCTGTACG | p426H7-wtILV2, p426H7-ILV2∆N54, p426H7-ILV2∆N85 |
| ILV2-C-Tag-r | AACTAATTACATGACTCGAGTCAGTGATGGTGATGGTGATGGTGCTTACCGCCTGTACGC | p426H7-ILV2wttag, p426H7-ILV2∆N54tag, p426H7-ILV2∆N85tag |
| Ilv5-wt-f | AACACAAAAACAAAAAGTTTTTTTAATTTTAATCAAAAAATGTTGAGAACTCAAGCCGC | p423H7-ILV5wt, p426H7-ILV5wttag |
| ILV5∆N48-f | AACACAAAAACAAAAAGTTTTTTTAATTTTAATCAAAAAATGAAGCAAATCAACTTCGGT | p423H7-ILV5∆N48, p426H7-ILV5∆N48tag |
| ILV5-r | GAATGTAAGCGTGACATAACTAATTACATGACTCGAGTTATTGGTTTTCTGGTCTCAAC | p423H7-ILV5wt, p423H7-ILV5∆N48 |
| ILV5-C-Tag-r | GAATGTAAGCGTGACATAACTAATTACATGACTCGAGTTAGTGATGGTGATGGTGATGTTGGTTTTCTGGTCTCAAC | p426H7-ILV5wttag, p426H7-ILV5∆N48tag |
| Ilv3-wt-f | AACACAAAAACAAAAAGTTTTTTTAATTTTAATCAAAAAATGGGCTTGTTAACGAAAGTTG | pRS42KH7-ILV3wt, pRS42KH7-ILV3wttag |
| ILV3∆N19DE-f | AACACAAAAACAAAAAGTTTTTTTAATTTTAATCAAAAAATGGATGAGGCAAAGAAGCTC | pRS42KH7-ILV3∆N19DE, pRS42KH7-ILV3∆N19DEtag |
| ILV3∆N19-f | AACACAAAAACAAAAAGTTTTTTTAATTTTAATCAAAAAATGGCAAAGAAGCTCAACAAG | pRS42KH7-ILV3∆N19, pRS42KH7-ILV3∆N19tag |
| ILV3∆N34-f | AACACAAAAACAAAAAGTTTTTTTAATTTTAATCAAAAAATGGGCCAAGGTGCGTCCCAG | pRS42KH7-ILV3∆N34, pRS42KH7-ILV3∆N34tag |
| ILV3∆N42-f | AACACAAAAACAAAAAGTTTTTTTAATTTTAATCAAAAAATGCTTTATGCCACCGGTTTC | pRS42KH7-ILV3∆N42, pRS42KH7-ILV3∆N42tag |
| ILV3∆N50-f | AACACAAAAACAAAAAGTTTTTTTAATTTTAATCAAAAAATGGAAGATTTCAAGAAGCCT | pRS42KH7-ILV3∆N50, pRS42KH7-ILV3∆N50tag |
| ILV3-r | GAATGTAAGCGTGACATAACTAATTACATGACTCGAGTCAAGCATCTAAAACACAACCG | pRS42KH7-ILV3wt, pRS42KH7-ILV3∆N19DE, pRS42KH7-ILV3∆N19, pRS42KH7-ILV3∆N34, pRS42KH7-ILV3∆N42, pRS42KH7-ILV3∆N50 |
| ILV3-C-Tag-r | GAATGTAAGCGTGACATAACTAATTACATGACTCGAGTCAGTGATGGTGATGGTGATGAGCATCTAAAACACAACCGTTG | pRS42KH7-ILV3wttag, pRS42KH7-ILV3∆N19DEtag, pRS42KH7-ILV3∆N19tag, pRS42KH7-ILV3∆N34tag, pRS42KH7-ILV3∆N42tag, pRS42KH7-ILV3∆N50tag |
| ARO10-f | AACACAAAAACAAAAAGTTTTTTTAATTTTAATCAAAAAATGGCACCTGTTACAATTGAAAA | p424H7-Aro10, pRS42NH7-ARO10 |
| ARO10-r | GAATGTAAGCGTGACATAACTAATTACATGACTCGAGCTATTTTTTATTTCTTTTAAGTGCC | p424H7-Aro10, pRS42NH7-ARO1 |
| KivD-f | TTTAATCAAAAAGTTAACATGCATCACCATCACCATCACTATACAGTAGGAGATTACC | p424H7-kivD |
| KivD-r | GAATGTAAGCGTGACATAACTAATTACATGACTCGAGTTATGATTTATTTTGTTCAGCAAATA | p424H7-kivD |
| ADH2-f | AACACAAAAACAAAAAGTTTTTTTAATTTTAATCAAAAAATGTCTATTCCAGAAACTCA | pRS42HH7-ADH2 |
| ADH2-r | CAATACTCATTAAAAAACTATATCAATTAATTTGAATTAACTTATTTAGAAGTGTCAACAACGTATCTACCAGC | pRS42HH7-ADH2 |
| ADH1-f | AACACAAAAACAAAAAGTTTTTTTAATTTTAATCAAAAAATGTCTATCCCAGAAACTCA | p426H7-Adh1 |
| ADH1-r | GAATGTAAGCGTGACATAACTAATTACATGACTCGAGTTATTTAGAAGTGTCAACAA | p426H7-Adh1 |
| ADH2-f | AACACAAAAACAAAAAGTTTTTTTAATTTTAATCAAAAAATGTCTATTCCAGAAACTCA | p426H7-Adh2 |
| ADH2-r | GAATGTAAGCGTGACATAACTAATTACATGACTCGAGTTATTTAGAAGTGTCAACAA | p426H7-Adh2 |
| ADH3-f | AACACAAAAACAAAAAGTTTTTTTAATTTTAATCAAAAAATGTTGAGAACGTCAACATT | p426H7-Adh3 |
| ADH3-r | GAATGTAAGCGTGACATAACTAATTACATGACTCGAGTTATTTACTAGTATCGACGA | p426H7-Adh3 |
| ADH4-f | AACACAAAAACAAAAAGTTTTTTTAATTTTAATCAAAAAATGTCTTCCGTTACTGGGTT | p426H7-Adh4 |
| ADH4-r | GAATGTAAGCGTGACATAACTAATTACATGACTCGAGTTAATATTCATAGGCTTTCT | p426H7-Adh4 |
| ADH6-f | AACACAAAAACAAAAAGTTTTTTTAATTTTAATCAAAAAATGTCTTATCCTGAGAAATT | p426H7-Adh6 |
| ADH6-r | GAATGTAAGCGTGACATAACTAATTACATGACTCGAGCTAGTCTGAAAATTCTTTGT | p426H7-Adh6 |
| SFA1-f | AACACAAAAACAAAAAGTTTTTTTAATTTTAATCAAAAAATGTCCGCCGCTACTGTTGG | p426H7-Sfa1 |
| SFA1-r | GAATGTAAGCGTGACATAACTAATTACATGACTCGAGCTATTTTATTTCATCAGACT | p426H7-Sfa1 |
| FMO1-1_1 | CAAGCGCGCAATTAACCCTCACTAAAGGGAACAAAAGCTGTTAATTAACGTAAAAAGGAATGTACTTG | p425-synthILV235 |
| FMO1-1_2 | CCGATTTCACTTTCTCATCCTTATATTTTTCCTGTGCGGATTTTCCAGATTC | p425-synthILV235 |
| FMO1-2_1 | GTATGCTATACGAAGTTATTAGGTGATATCAGATCCACTAGTGGAGGCCATCCTTTTAAAGATC | p425-synthILV235 |
| FMO1-2_2 | GGAACCCTAAAGGGAGCCCCCGATTTAGAGCTTGACGGCGCGCCGATTTTCCTCAGTCCCTTAGG | p425-synthILV235 |
| loxP-1 | CGAAAATTCTGCGTTCGTTAAAGCTTTCGAGAAGGATATTATTTGCTGAAGCTTCGTACGCTGC | p425-synthILV235 |
| loxP-2 | CCACTAGTGGATCTGATATC | p425-synthILV235 |
| FMO1-PFK1p-f | GTAAAAAAAAAAGAAAACTTTCTTTTTGAATCTGGAAAATCCGCACAGGAAAAATATAAG GATGAGAAAGTGAAATC | p425-synthILV235 |
| PFK1p-Ilv3∆N48-r | CTTTGATATGATTTTGTTTCAGATTTTTTATA | p425-synthILV235 |
| Ilv3∆N48-f | AGCTTTTATATAAAAAATCTGAAACAAAATCATATCAAAGATGGCTAAGAAGTTGAACAA | p425-synthILV235 |
| Ilv3∆N48-r | AATACTCATTAAAAAACTATATCAATTAATTTGAATTAACTTAAGCGTCCAAAACACAAC | p425-synthILV235 |
| Ilv3∆N48-FBA1t-f | GGTTTCTAACGCTTCTAACGGTTGTGTTTTGGACGCTTAAGTTAATTCAAATTAATTGATATAGTTTTTTAATGAG | p425-synthILV235 |
| FBA1t-HXT7p-r | CAGAAGAACACGCAGGGGCCCGAAATTGTTCCTACGAGCATGAGCTATCAAAAACGATAGATCGATTAG | p425-synthILV235 |
| HXT7p-f | GCTCGTAGGAACAATTTCGGGCC | p425-synthILV235 |
| HXT7p-Ilv2∆N54-r | TTTTTGATTAAAATTAAAAAAACTTTTTGTTTT | p425-synthILV235 |
| HXT7p-Ilv2∆N54-f | AAACACAAAAACAAAAAGTTTTTTTAATTTTAATCAAAAAATGCCAGAACCAGCTCCATC | p425-synthILV235 |
| Ilv2∆N54-CYC1t-r | CGTGAATGTAAGCGTGACATAACTAATTACATGACTCGAGTTAGTGCTTACCACCGGTT | p425-synthILV235 |
| CYC1t-f | CTCGAGTCATGTAATTAGTTATGTCACGC | p425-synthILV235 |
| CYC1-r | AAATTAAAGCCTTCGAGCGTCC | p425-synthILV235 |
| CYC1t-FBA1p-f | TTGCTTGAGAAGGTTTTGGGACGCTCGAAGGCTTTAATTTTGGGTCATTACGTAAATAAT | p425-synthILV235 |
| FBA1p-r | TTTGAATATGTATTACTTGGTTATGGTTATAT | p425-synthILV235 |
| FBA1p-Ilv5∆N48-f | TTTGTCATATATAACCATAACCAAGTAATACATATTCAAAATGAAGCAAATTAACTTCGG | p425-synthILV235 |
| Ilv5∆N48-PGK1t-r | GAGAAAAGAAAAAAATTGATCTATCGATTTCAATTCAATTCAATTTATTGGTTTTCTGGTCTCAACTTTCT | p425-synthILV235 |
| PGK1-f | ATTGAATTGAATTGAAATCGATAGATCAATT | p425-synthILV235 |
| PGK1-r | AAATAATATCCTTCTCGAAAGCTTTAACGA | p425-synthILV235 |
| Del-ilv2-f | GAGCTAAGAGGAGATAAATACAACAGAATCAATTTTCAATTCGTACGCTGCAGGTCGAC | Isoy8, Isoy16 |
| Del-ilv2-r | TTTTTACTGAAAATGCTTTTGAAATAAATGTTTTTGAAATGCATAGGCCACTAGTGGATC | Isoy8, Isoy16 |
| Del-ilv3-f | TTCTTGTATTTTTTTGTAAACAGCCAAGAAAAAAGTAGAGTTCGTACGCTGCAGGTCGAC | Isoy10, Isoy16 |
| Del-ilv3-r | ATCTCTATATATATATTCATCGATTGGGGCCTATAATGCAGCATAGGCCACTAGTGGATC | Isoy10, Isoy16 |
| Del-ilv5-f | ATTTTTTTACCCTACCAGCAATATAAGTAAAAAATAAAACTTCGTACGCTGCAGGTCGAC | Isoy12, Isoy16 |
| Del-ilv5-r | CAAGAGAAAAAGTTTCCAGCACTTGATATTATTTTCCTCTGCATAGGCCACTAGTGGATC | Isoy12, Isoy16 |
